# Feasibility of Recruiting People with Alzheimer’s Disease and Related Dementias in Healthcare Research

**DOI:** 10.1093/geroni/igaf122.2223

**Published:** 2025-12-31

**Authors:** Rashmita Basu, Donna Roberson, Rodriguez Gonzalez, Shivrajo Patil

**Affiliations:** East Carolina University, Greenville, North Carolina, United States; East Carolina University, Greenville, North Carolina, United States; East Carolina University, Greenville, North Carolina, United States; East Carolina University, Greenville, North Carolina, United States

## Abstract

With Alzheimer’s Disease and Related Dementia (ADRD) increasing in the U.S., more research is needed to deliver patient-centered care. However, recruiting people with ADRD is challenging—this pilot study aimed to test the feasibility of collecting self-reported health data from people with ADRD. The primary data collection site was a geriatric clinic in eastern North Carolina. Potential patients were identified based on dementia diagnoses from the electronic medical record system (EPIC). After the clinic visits with the healthcare providers, we consented to those with “mild” or “moderate” dementia using a short portable mental status questionnaire. Participants self-reported (in person or over the phone) survey questions focused on their health and well-being. Thirty-five patients with ADRD consented to the study, and 25 participants completed the survey (>70% completion rate). The sample average age was 71; 62% were female, 42% were White, 56% were African American, and 54% had limitations in at least one daily activity. Participants were satisfied with their life circumstances, with an average score of 4.5 (range 0-5). Qualitative interviews indicated the need for improvements in dementia care, including better person-centered care and resources. None expressed difficulty with the survey completion. Although recruiting people with ADRD for healthcare research is challenging, it can be done. Partnering with healthcare providers and community support groups was an effective recruitment strategy. Our results indicate that community-dwelling individuals with ADRD can complete the surveys to report their health and well-being. Self-reporting was feasible and should be used in research to design person-centered care.

